# Isolated congenital hepatic fibrosis associated with *TMEM67* mutations: report of a new genotype–phenotype relationship

**DOI:** 10.1002/ccr3.981

**Published:** 2017-05-23

**Authors:** Ida Vogel, Peter Ott, Dorte Lildballe, Stephen Hamilton‐Dutoit, Hendrik Vilstrup, Henning Grønbæk

**Affiliations:** ^1^ Department of Clinical GeneticsAarhus University HospitalAarhusDenmark; ^2^ Department of Hepatology and Gastroenterology VAarhus University HospitalAarhusDenmark; ^3^ Department of PathologyAarhus University HospitalAarhusDenmark

**Keywords:** Ciliopathy, congenital hepatic fibrosis, meckelin, *TMEM67*

## Abstract

We report an otherwise healthy 32‐year‐old man with portal hypertension, variceal bleeding, and congenital hepatic fibrosis with ductal plate malformation. Genetic screening identified two TMEM67 mutations. Biallelic TMEM67 mutations are known to cause Joubert/Meckel syndrome or nephronopthisis with hepatic fibrosis, but have never been found in isolated hepatic fibrosis.

## Introduction

Congenital hepatic fibrosis is a heterogeneous disease entity that may occur either sporadically or as a part of several recessive malformation syndromes – the so‐called ciliopathies – due to defective cilia. The primary cilium, the cell's “antenna,” is important for cell orientation and for the ability to receive signaling molecules, making it essential for embryonic development 1. Mutations in genes coding for components of the primary cilium underlie development of many ciliopathies 2, a group of disorders known for their considerable genetic and phenotypical heterogeneity. Congenital hepatic fibrosis is seen within a subgroup of ciliopathies characterized by so‐called fibrocystic liver diseases, which to a variable degree also include polycystic liver and kidneys, choledochal cysts, Caroli's disease, and biliary microhamartoma. One rare but important member of this group is the autosomal recessive Meckel syndrome that is characterized by encephalocele, polydactyly, cystic kidneys, and liver fibrosis. *TMEM67*, the gene coding for meckelin, is one of the genes that can be mutated in this syndrome. However, *TMEM67* mutations are also known to cause a continuum of recessive conditions that include Joubert syndrome, COACH (cerebellar vermis hypoplasia, oligophrenia, congenital ataxia, coloboma, and hepatic fibrosis) syndrome, nephronopthisis, and Bardet–Biedl syndrome 3. While these have very different phenotypes with extrahepatic malformations, they also include congenital hepatic fibrosis.

We report an otherwise healthy 32‐year‐old man with portal hypertension and variceal bleeding whose liver biopsy showed congenital hepatic fibrosis with ductal plate malformation. He had biallelic TMEM67 mutations and thereby represents a new genotype/phenotype combination characterized by isolated congenital hepatic fibrosis.

## Case History

We describe a 32‐year‐old male with congenital hepatic fibrosis, portal hypertension, and esophageal varices. He is currently treated with antibiotics for acute cholangitis and a newly developed portal vein thrombosis, where the patient is awaiting stent treatment.

In 2006, he presented with esophageal variceal bleeding. At the local hospital, he was diagnosed with splenic enlargement and underwent a splenectomy to exclude malignant hematological disease. In 2006, he was referred to our institution for further evaluation and treatment due to the presence of varices without a specific cause for liver disease.

At referral, biochemistry showed elevated liver enzymes such as alanine aminotransferase 121 U/L (normal range 10–70 U/L), alkaline phosphatase 560 (35–105 U/L), *γ*‐glutamyl transferase 731 U/L (10–80 U/L), while bilirubin (10 *μ*mol/L (5–25 *μ*mol/L), international normalized ratio (INR), and hematological and renal functions were normal. The diagnostic workup excluded common causes of chronic liver disease. There were normal immunoglobulins (A, G, M, and IgG subclasses), negative tests for autoantibodies (smooth muscle cell, antimitochondria, antinuclear, and antineutrophil cytoplasmic), and negative viral hepatitis (A, B, C) and HIV tests. Serum ferritin, coerulopasmin, and alpha‐1‐antitrypsin were normal. He was found to be heterozygous for a Factor V Leiden mutation with an increased risk for thrombotic events and is now on oral anticoagulation. Besides the current thrombosis of the portal vein, no other thrombotic events have occurred. Liver biochemistry was stable over time. Metabolic liver function was assessed annually from 2007 to 2014 by galactose elimination capacity 4 and showed stable liver function at around 70% of expected values. Pruritus was treated with ursodeoxycholic acid and cholestyramine, but most effectively with rifaximin. Esophageal varices were treated by endoscopic band ligation and *β*‐blockers, after which he was free of variceal bleeding. A liver vein catheterization showed a hepatic gradient of 20 mmHg with a reduction to 14 mmHg by *β*‐blockade.

Diagnostic imaging included magnetic resonance (MR) and computed tomography (CT) scanning and MR cholangiopancreatography. CT scans in 2007 and 2014 showed liver enlargement with hypoplasia of the right liver lobe and compensatory enlargement of the left lobe. There was no portal vein thrombosis. Kidneys were of normal size. There were no hepatic or kidney cysts.

**Figure 1:**
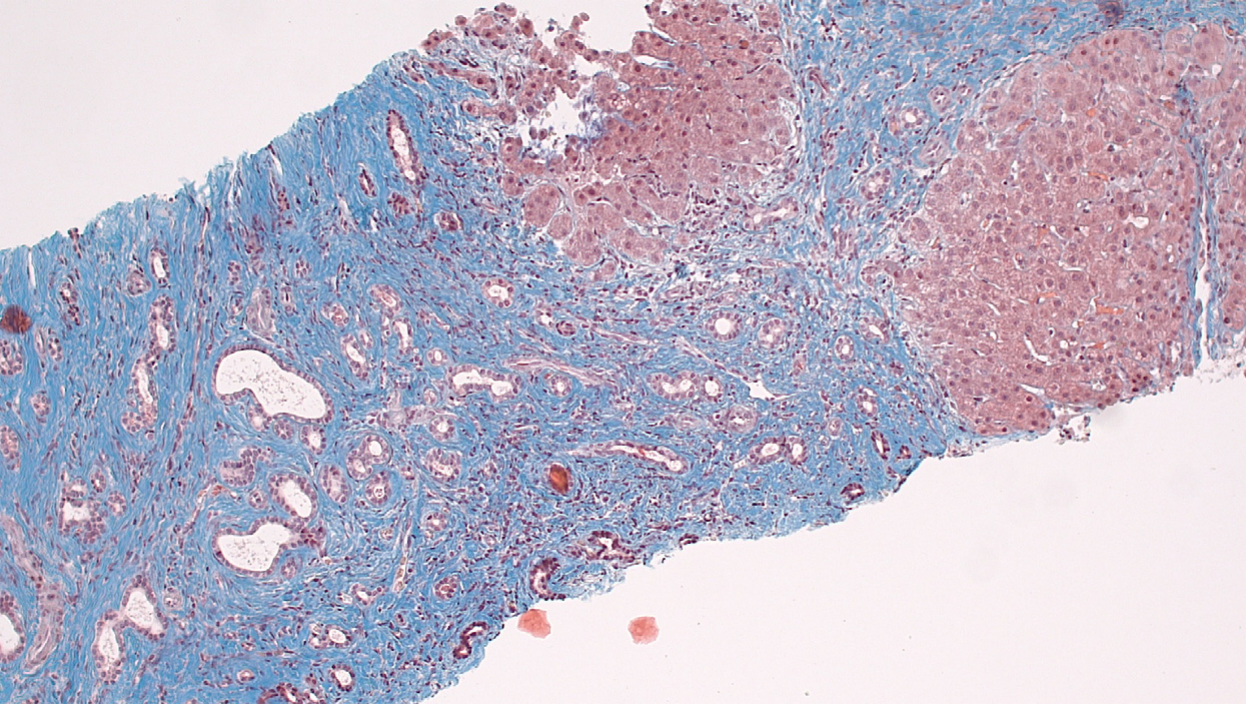
Core needle biopsy from the patient's liver showing congenital hepatic fibrosis. There is septal bridging fibrosis separating irregular nodules of liver cells with partial retention of lobular architecture. The fibrotic septa contain numerous irregular, often dilated biliary structures, focally with features of ductal plate malformation (Masson's trichrome).

An initial liver biopsy (2006, suboptimal size) showed liver fibrosis with septa, preserved interlobular bile ducts with nonspecific bile duct proliferation, and mild septal inflammation. A new liver biopsy (2015) showed distinct architectural changes with broad areas of periportal septal bridging fibrosis separating irregular nodules of benign‐looking hepatocytes with partial retention of lobular architecture (Fig. 1). The fibrotic septa contained large numbers of abnormal, irregular, often slightly dilated biliary structures, focally with an appearance reminiscent of ductal plate malformation. There was only minimal inflammation, without cholangitis and with no cholestasis. The histological changes were consistent with congenital hepatic fibrosis.

**Figure 2:**
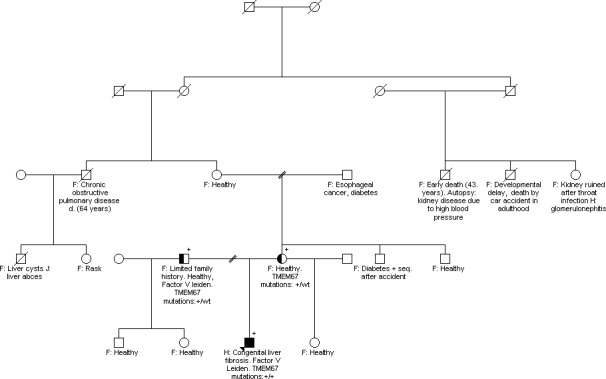
Family pedigree. A detailed family history revealed only one affected family member (the proband) with congenital hepatic fibrosis. No other ciliopathy‐related diseases were found. Only the proband and his parents were tested for the *TMEM67* mutations (*TMEM67* [NM_153704.5]: [622A>T];[641A)G])). F: Family Information; H: Histology.

Because of his unusual presentation and histological findings, the patient was referred for a genetic workup including family anamnesis, drawing of a pedigree and genetic testing. He was born full term after an uncomplicated pregnancy, and there were no neonatal complications. He had reached developmental milestones at the expected ages and showed no increased frequency of common childhood diseases. He completed public school and secondary school without intellectual, physical or social problems, and participated in normal activities. A pedigree demonstrated no accumulation in the patients' family of liver or kidney disease, eye or hearing problems, intellectual disability, or congenital malformations in the patient's family (Fig. 2). The parents are not consanguineous and of Danish and British origin. Testing for rare genetic liver diseases was performed on DNA derived from whole blood using targeted panels for liver diseases and for cystic kidney/liver diseases. Two mutations in *TMEM67* were found (*TMEM67* [NM_153704.5]: [622A>T];[641A>G]). Parental analyses confirmed that these mutations were on different alleles. The maternally inherited mutation, c.622A>T, was previously reported and associated with Meckel syndrome 5. Prediction programs (MutationTaster) indicate that this mutation will cause a premature stop codon p.(Arg208Ter) and that is likely to be disease causing. The CADD score is 33 6. The minor‐allele frequency of the variant is 0.014% of the ExAc database. Prediction programs (MutationTaster, Provean, Sift, Polyphen) uniformly predict that the paternally inherited mutation, c.641A>G mutation, will cause a change in amino acid p.(Tyr214Cys) and that it is likely to damage the protein function. The Grantham score of the change is 194. The CADD score is 26. The minor‐allele frequency of the variant is 0.0025% of the ExAc database. Thus, the patient was compound heterozygote for one truncating and one missense mutation in *TMEM67*. The patient was subsequently re‐examined for signs of Meckel–Gruber or Joubert syndrome, but no additional signs of these syndromes were found at the physical examination besides congenital hepatic fibrosis.

## Material and Methods

The patient's DNA was screened for mutations known to underlie a spectrum of liver and cystic diseases using two extensive in‐house‐targeted gene panels screening for mutations in more than 150 genes. Average coverages of 448 and 491, respectively, were achieved with >98% of bases being covered >30x. Genomic DNA was isolated from peripheral blood leukocytes using the magnetic bead‐based automated Chemagic MSM1 instrument, following the manufacturer's instructions (PerkinElmer Chemagen, Baesweiler, Germany). A library for Illumina paired‐end sequencing was constructed from 1 *μ*g genomic DNA using the KAPA HTP Library Preparation Kit according to the manufacturer's instructions (KAPA Biosystems Inc., Wilmington, MA). The generated libraries were enriched for regions of interest using a customized targeting probe set (SeqCap EZ Choise, Roche Nimblegen, Inc., Madison, WI) and sequenced on a MiSeq Desktop Sequencer (Illumina, San Diego, CA). The reads obtained from sequencing were aligned to the human genome (hg19), and variants were called using Biomedical Genomics Workbench v.2 (CLC bio‐Qiagen, Aarhus, Denmark).

## Discussion

We describe the first case of isolated congenital hepatic fibrosis caused by *TMEM67* mutations, one truncating and one missense mutation. *TMEM67* mutations have been identified as a likely cause of multi‐organ syndromes associated with congenital hepatic fibrosis 3, 7. However, this 32‐year‐old man demonstrates none of the other findings associated with the Joubert, Meckel, COACH, nephronopthisis, or Bardet–Biedl syndromes also caused by *TMEM67* mutations.

We suggest it is relevant to screen patients presenting with unexplained portal hypertension with suspected congenital hepatic fibrosis systematically for gene abnormalities involved in ciliopathies including *TMEM67*, even when they exhibit only limited clinical abnormalities.

Most cells have one immotile primary cilium that is essential for cell organization and positioning, defective cilia leading to malformations 1. In ciliary dysfunction – the so‐called ciliopathies – many of the more than 30 proteins implicated in this disease, localize to the transition zone, which is a junction site between the primary cilium and the basal body 8. Meckelin is one of these proteins involved in the molecular architecture of the transition zone 9, and *TMEM67*, the gene coding for meckelin, is one of the genes that can be mutated in ciliopathies. Previously, meckelin expression has been demonstrated in early human embryos using in situ hybridization in kidney, liver, retina, brain, and developing limbs 10. Hepatic expression was primarily reported in the biliary epithelium of larger bile ducts, while cytoplasmic staining of hepatocytes could not be shown with certainty 10.

Although meckelin dysfunction was originally only thought to cause Meckel syndrome, it is now also recognized in a variety of phenotypes within the spectrum of the Joubert, Joubert‐related, COACH, nephronopthisis, and Bardet–Biedl syndromes, with the great majority manifesting congenital hepatic fibrosis accompanied by multiple other malformations 3, 7, 11. In patients with nephronopthisis and hepatic fibrosis, Otto et al. (2009) identified homozygous or compound heterozygous missense mutations in *TMEM67* 11. Mutations in the *TMEM67* gene were not found in any of 105 patients with nephronopthisis without liver fibrosis, suggesting that hepatic fibrosis is a specific feature of *TMEM67* mutations. How *TMEM67* mutations cause congenital hepatic fibrosis is unknown, but could result from defective cilia in the biliary tubules leading to ductal plate malformation 12, in particular, because defective cilia are known to cause proliferation and cyst formation in polycystic kidney disease 1, 12.

Otto et al. 11 hypothesized that the severity of mutations was dependent on whether there were truncating mutations with a complete loss‐of‐function (Meckel, COACH, and Joubert syndromes) or missense mutations with decreased function, but with retention of some expression in the milder spectrum (nephronopthisis). However, our patient with a mild phenotype seems to contradict this hypothesis as he has one truncating and one missense mutation. We hypothesize that in this case/our patient, protein is still expressed from one allele (the missense allele) and that this is enough to only cause a mild phenotype compared to previously published results describing homozygote loss‐of‐function mutations.

Meckelin consists of an extracellular N‐terminal domain, three to seven transmembranous regions and an intracellular C‐terminal domain 13. Both mutations in our patient are found in the extracellular domain of the protein immediately after a cysteine‐rich region. Mutations in this area could potentially be less severe than mutations occurring in either the transmembranous, or in the nearest extracellular regions, particularly as these harbors many of the previously reported mutations 3, 5, 14.

The natural history of congenital hepatic fibrosis is variable, generally being clinically most severe when presenting in utero, or during childhood and adolescence. In surviving adults, the prognosis is usually good, although as in our patient, progressive fibrosis may result in portal hypertension with variceal bleeding. The cause of noncirrhotic portal hypertension is elusive in a proportion of young patients. Clinically, our case emphasizes that isolated liver fibrosis in some of these patients may have a genetic basis. We suggest it is relevant to screen patients presenting with unexplained portal hypertension with suspected congenital hepatic fibrosis systematically for gene abnormalities involved in ciliopathies including *TMEM67*, even when they exhibit only limited clinical abnormalities.

## Conflict of Interest

None of the authors have any conflict of interests.

## Authorship

IV: Genetic counseling and pedigree. DL: Genetic testing. SHD: Histology. HG, PO and HV: patient care. All have contributed to and accepted the final version of the manuscript. IV and HG: received a clinical research grant from the NOVO Nordisk Foundation, and HG: obtained research funding from “Savværksejer Jeppe Juhl og hustru Ovita Juhls mindelegat”, Abbvie, IPSEN, and Novartis.
